# Frequent Gastrointestinal Cancer Complications in Japanese Patients with Acute or Chronic Coronary Syndrome Undergoing Percutaneous Coronary Intervention

**DOI:** 10.3390/jcm14061807

**Published:** 2025-03-07

**Authors:** Yasuyuki Chiba, Shogo Imagawa, Yuki Takahashi, Kimitoshi Kubo, Kenta Otsuka, Kyo Shimazu, Teisuke Anzai, Kazuya Yonezawa, Mototsugu Kato, Toshihisa Anzai

**Affiliations:** 1 Division of Cardiology, National Hospital Organization Hakodate Medical Center, Hakodate 041-8512, Japan; imagawa.shogo.pd@mail.hosp.go.jp (S.I.); kyokui080027@gmail.com (Y.T.); kenta.sapmed@gmail.com (K.O.); shimazu.kyo.te@mail.hosp.go.jp (K.S.); anzai.teisuke.uj@mail.hosp.go.jp (T.A.); yonezawa.kazuya.td@mail.hosp.go.jp (K.Y.); 2 Division of Gastroenterology, National Hospital Organization Hakodate Medical Center, Hakodate 041-8512, Japan; kubo.kimitoshi.cx@mail.hosp.go.jp (K.K.); mkato1957@gmail.com (M.K.); 3 Department of Gastroenterology, Public Interest Foundation Hokkaido Cancer Society, Sapporo 065-0026, Japan; 4 Department of Cardiovascular Medicine, Faculty of Medicine and Graduate School of Medicine, Hokkaido University, Sapporo 060-8638, Japan; anzai@med.hokudai.ac.jp

**Keywords:** dual antiplatelet therapy, percutaneous coronary intervention, gastrointestinal malignancy, bleeding, gastrointestinal endoscopy

## Abstract

**Background/Objective:** Gastrointestinal bleeding is a major complication of dual antiplatelet therapy (DAPT) in patients undergoing percutaneous coronary intervention (PCI). Malignancy may be detected due to gastrointestinal bleeding, necessitating critical decisions regarding treatment selection and influencing patient prognosis. **Methods:** This single-center, retrospective, observational study included 501 Japanese patients who underwent initial PCI between January 2019 and January 2023. Of these patients, 393 who underwent perioperative upper and lower gastrointestinal endoscopy were evaluated for the presence of gastrointestinal malignancy. **Results:** Of the total patients, 36% presented with acute coronary syndrome (ACS). Gastrointestinal malignancies were identified in 30 patients (8%), including 18 cases of colorectal cancer and eight cases of gastric cancer. No difference in the frequency of malignancies was observed between patients with ACS and chronic coronary syndrome (CCS) (*p* = 0.7398). Malignancies were significantly more common in patients with positive fecal immunochemical testing (FIT) (*p* < 0.0001); however, FIT did not detect all malignancies. The 1500-day survival rate for patients with gastrointestinal malignancies was 64%, with no difference in overall survival between treatment modalities. **Conclusions:** A considerable proportion of Japanese patients undergoing PCI had gastrointestinal malignancies, regardless of whether they had ACS or CCS, and their prognosis was poor. Gastrointestinal endoscopic evaluation in the perioperative period of PCI could detect malignancy without complications and might lead to appropriate cancer treatment.

## 1. Introduction

The incidence of cardiovascular diseases is increasing worldwide owing to population growth and aging societies. Among these, ischemic heart disease (IHD) is the most common and is a leading cause of death [1,2]. Although recent advances in treatment, particularly percutaneous coronary intervention (PCI), have improved prognosis, mortality remains high, especially in cases of out-of-hospital cardiac arrest, where increased adrenaline dosage is associated with increased mortality [3], and early PCI is important in such cases with acute myocardial infarction.

Dual antiplatelet therapy (DAPT) is required as a standard drug therapy during the perioperative period of PCI. Since DAPT significantly reduces the risk of stent thrombosis but increases the risk of bleeding complications, multiple lines of evidence support a shorter duration of DAPT [4,5,6,7].

Gastrointestinal bleeding is a major complication of DAPT that affects patient prognosis [8]. In patients with acute myocardial infarction taking antiplatelet therapy, gastrointestinal bleeding is associated with a new cancer diagnosis [9], necessitating critical decisions regarding treatment selection. An increased risk of cardiac death and bleeding complications has also been reported in cancer patients undergoing PCI, particularly in patients diagnosed with cancer within a year of the procedure [10].

Fecal immunochemical testing (FIT) is a commonly used screening tool for gastrointestinal bleeding, particularly for colorectal cancer [11,12], and is often employed clinically to assess the risk of gastrointestinal bleeding before PCI. However, FIT has known limitations in detecting gastrointestinal malignancies.

Therefore, this study investigated the frequency of gastrointestinal malignancies, associated factors, and prognoses in patients undergoing PCI, utilizing upper and lower gastrointestinal endoscopy.

## 2. Materials and Methods

### 2.1. Study Protocol and Population

This was a retrospective, observational study conducted at a single institution in Japan. Some patient data from this study have recently been submitted (Higashino M, MD, PhD, Hokkaido University, Sapporo, Japan, unpublished data, 2025); however, this study aimed to demonstrate the utility of perioperative upper and lower gastrointestinal endoscopy in patients undergoing PCI, specifically to evaluate differences in the frequency of gastrointestinal malignancies in acute coronary syndrome (ACS) and chronic coronary syndrome (CCS) and to assess prognosis after malignancy detection.

**Figure 1:**
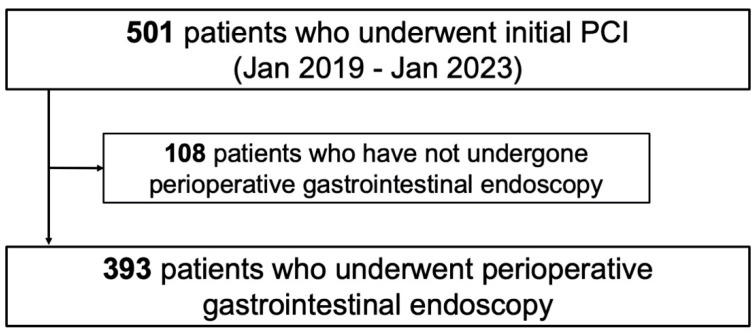
**Patient selection.** PCI, percutaneous coronary intervention.

A total of 501 hospitalized Japanese patients who underwent initial PCI for IHD, including ACS, were screened between January 2019 and January 2023. Of these, 108 patients who did not undergo either upper or lower gastrointestinal endoscopy during the perioperative period of PCI because they did not consent to endoscopy or because their general condition precluded such an examination were excluded. The perioperative period was defined as early post-PCI during hospitalization for patients with ACS, and within 12 months before PCI for patients with CCS. This definition is due to the most important objective of this study, which is to detect gastrointestinal malignancies that may affect the survival of patients undergoing PCI and to prevent bleeding complications from antiplatelet therapy. Consequently, 393 patients were included in the final analysis (Figure 1). Patient information and clinical course data were collected from the hospital database. The study protocol was approved by the Institutional Review Board of National Hospital Organization Hakodate Medical Center (No. 1105002), and patients were informed of their right to opt out of participation via the hospital’s homepage.

### 2.2. DAPT Administration and Gastrointestinal Examination

All patients with ACS received a loading dose of DAPT prior to emergency revascularization, and the maintenance dose of DAPT was continued the following day. FIT and upper or lower gastrointestinal endoscopy were performed during hospitalization for the detection of hemorrhagic lesions or malignancies in the upper or lower gastrointestinal tract, and if malignancy was detected, the duration of DAPT was determined according to its progression and bleed risk.

In patients with CCS, the dosage and timing of the antiplatelet agents, as well as the timing of FIT and endoscopy, were left to the discretion of the attending physicians, but as a rule, regular doses of antiplatelet agents were introduced after evaluation or appropriate treatment for malignancy. In patients with concomitant atrial fibrillation/flutter and who were on anticoagulation therapy, triple therapy was limited to a short period during hospitalization, in accordance with the current evidence. All patients were prescribed the appropriate doses of aspirin and prasugrel for DAPT.

### 2.3. Statistical Analysis

The Shapiro–Wilk test was used to assess the normality of continuous variables. Continuous variables are expressed as mean ± standard deviation or as median and interquartile range, and comparisons were made using either an unpaired Student’s *t*-test or a Wilcoxon rank–sum test, as appropriate. Categorical variables are expressed as numbers (percentages) and were compared between groups using the chi-square test.

To identify the independent determinants of malignancy detection, multivariate logistic regression analyses were performed using variables with a *p*-value < 0.05 in the univariable analyses. Kaplan–Meier analysis and the log-rank test were performed to assess overall survival. A *p*-value < 0.05 was considered significant for all tests. All statistical analyses were performed using JMP software (version 17.0; SAS Institute Inc., Cary, NC, USA).

## 3. Results

### 3.1. Patient Characteristics

A total of 501 patients were screened, and 393 were enrolled in this study, as shown in Figure 1. The patient characteristics are summarized in Table 1. The age of the patients was 75 (66–82) years, and 277 patients (70%) were male. Among the cohort, 36% had ACS and 23% had heart failure. Gastrointestinal malignancies were found in 30 patients (8%). Atrial fibrillation/flutter was present in 68 (17%) of all patients and in 7 (23%) of patients with malignancy; all of them were on anticoagulation therapy. More than half of patients had a history of smoking. All patients underwent revascularization using drug-eluting stents. None of the patients had been treated or diagnosed with gastrointestinal malignancies prior to the targeted endoscopy.

### 3.2. Association Between Patients with PCI and Malignancy

**Figure 2:**
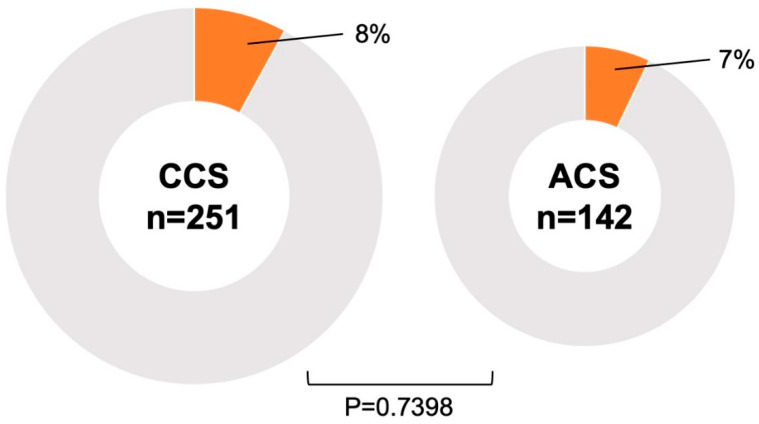
**Association between ACS/CCS and malignancy.** There was no difference in the frequency of malignancy between patients with ACS and CCS. ACS, acute coronary syndrome; CCS, chronic coronary syndrome.

**Figure 3:**
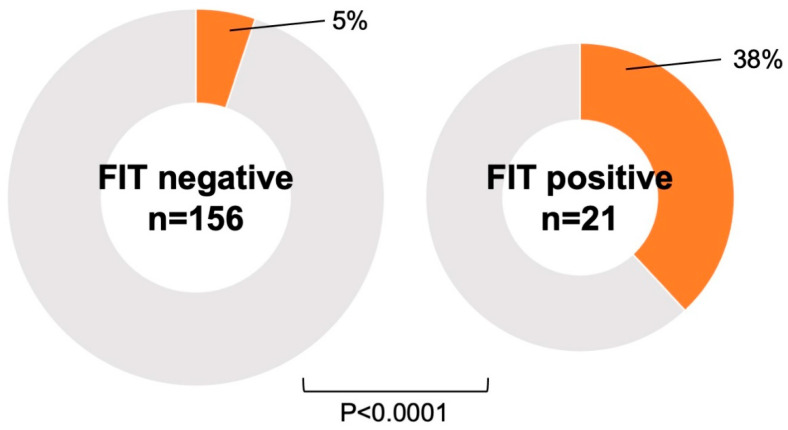
**Association between FIT and malignancy.** Gastrointestinal malignancies were significantly more frequent in cases with positive FIT. FIT, fecal immunochemical testing.

**Figure 4:**
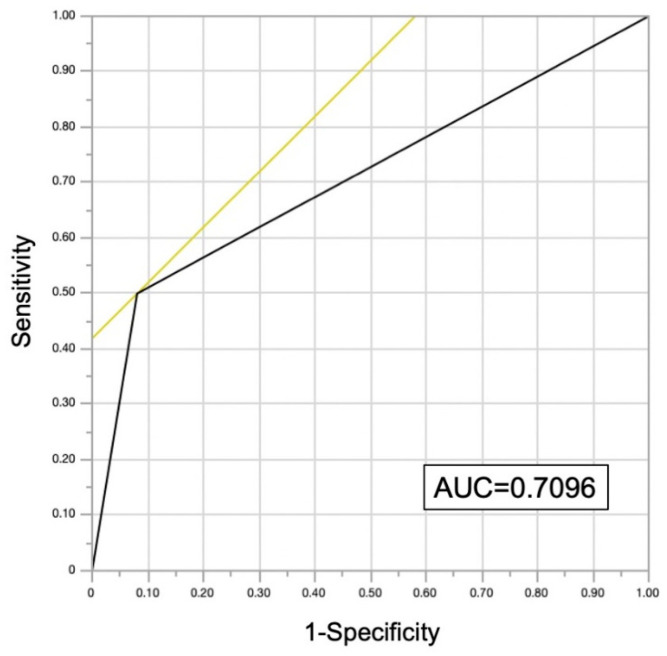
**The receiver operating characteristic analysis for detection of gastrointestinal malignancies by FIT.** AUC, area under the curve; FIT, fecal immunochemical testing.

Gastrointestinal malignancies were found in 30 patients (8%), including 18 cases of colorectal cancer, 8 cases of gastric cancer, 2 cases of esophageal cancer, 1 case of laryngeal cancer, and 1 overlapping case of gastric and colorectal cancer. All of these patients had malignancy detected incidentally by targeted endoscopy rather than by bleeding resulting from PCI or antiplatelet therapy. Twelve patients were treated endoscopically, and eleven were treated surgically. Table 2 shows the progression of the malignancy and the treatments performed. The interval between cancer detection by endoscopy and PCI in the 30 patients with malignancy was 22.5 (7.8–74.3) days. There was no difference in the frequency of malignancy between patients with ACS and CCS (*p* = 0.7398) (Figure 2). FIT was performed in 177 cases without dietary or medicinal restrictions prior to endoscopy, and malignancies were significantly more frequent in cases with positive FIT results (*p* < 0.0001) (Figure 3). The sensitivity and specificity of FIT for detecting malignancy were 50% and 92%, respectively. The receiver operating characteristic analysis for detection of gastrointestinal malignancies by FIT is shown in Figure 4. The area under the curve was 0.7096.

In the non-cancer patients (n = 363), gastric ulcers and benign tumors such as polyps without major bleeding were found in some cases.

**Figure 5:**
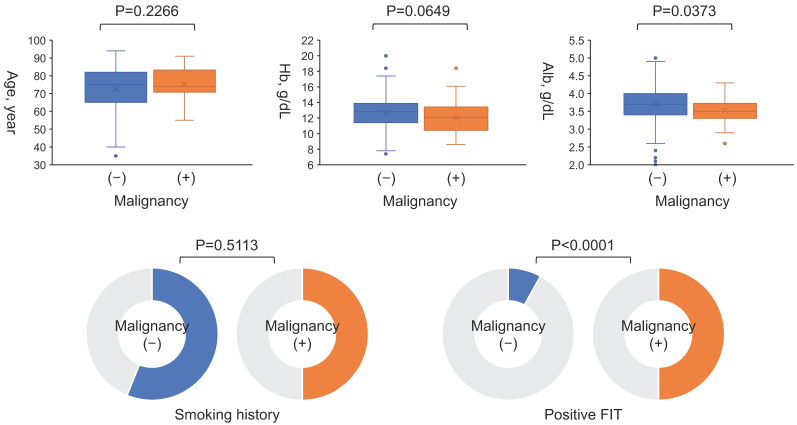
**Association of each parameter with malignancy.** Albumin level and FIT were significantly associated with malignancy, but not age and smoking history. Hemoglobin levels were not statistically significantly different, but they were lower in patients with malignancy. FIT, fecal immunochemical testing.

The association between malignancy and each parameter is shown in Figure 5. Age and smoking history were not associated with malignancy; however, albumin level and FIT were significantly associated with malignancy. Hemoglobin levels were not statistically significantly different, but they were lower in patients with malignancy (*p* = 0.0649).

In the multivariate analysis for malignancy detection, only FIT results were significantly associated with malignancy (Table 3).

### 3.3. Survival of Patients with PCI with Gastrointestinal Malignancies

During a median follow-up period of 1260 (796–1500) days, 10 patients with gastrointestinal malignancies died. The causes of death were myocardial infarction in one case, gastrointestinal malignancy in four cases, cancer treatment-related infection in two cases, anemia in one case, and unknown causes in two cases. Myocardial infarction death occurred in the acute phase of the disease, and most of the other deaths were malignancy related.

**Figure 6:**
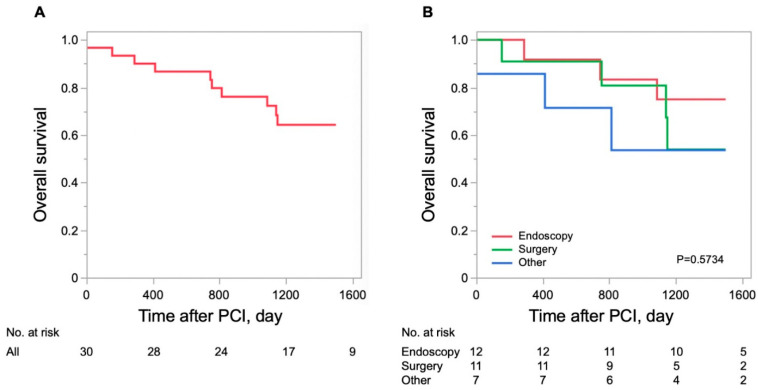
**Kaplan–Meier curves for overall survival.** (**A**) A survival curve for all patients with gastrointestinal malignancies. The survival rate at 1500 days was 64% in patients with gastrointestinal malignancies. (**B**) Survival curves for patients with malignancies by treatment. There was no difference in overall survival rate among the surgical, endoscopic, and other treatment groups (chemotherapy, radiation therapy, and best supportive care).

Figure 6 shows the survival curves for patients with detected gastrointestinal malignancies. The survival rate at 1500 days was 64% for patients with gastrointestinal malignancies detected in the perioperative period of PCI. There was no difference in overall survival rate among the surgical, endoscopic, and other treatment groups (chemotherapy, radiation therapy, and best supportive care).

## 4. Discussion

The most important objective of this study was to detect gastrointestinal malignancies that might affect survival in patients undergoing PCI and to prevent bleeding complications due to antiplatelet therapy. In accordance with this objective, we defined the peri-PCI period as early during hospitalization after PCI in patients with ACS and before (within 1 year) scheduled PCI in patients with CCS, and we included patients who underwent gastrointestinal endoscopy during this period. The main findings of this study are as follows: (1) gastrointestinal malignancies were present in 8% of PCI cases, and their prognosis was poor; (2) there was no difference in the frequency of gastrointestinal malignancies between patients with ACS and CCS; (3) positive FIT results were associated with the presence of gastrointestinal malignancy, but it did not detect all patients with malignancy. This was a clinically relevant study that performed both upper and lower gastrointestinal endoscopy in the perioperative period of PCI, whether for ACS or CCS, and examined the presence of gastrointestinal malignancies.

### 4.1. Coronary Artery Disease and Gastrointestinal Cancer

Cardiovascular disease and cancer remain the two leading causes of death in developed countries, despite advances in their diagnosis, prevention, and treatment. Importantly, several classical risk factors and underlying pathophysiological mechanisms associated with cardiovascular disease are also linked to an increased risk of cancer [13]. Chan et al. reported that 4.4% of patients with at least 50% stenosis in the major coronary arteries had colorectal cancer, significantly more than in the general population, and that metabolic syndrome and smoking were associated with advanced colorectal lesions and coronary artery disease [14]. On the other hand, few reports have clarified the relationship between gastric cancer and IHD; however, it has been suggested that *Helicobacter pylori*, which induces a high rate of gastric cancer, may also induce an inflammatory response that may lead to coronary artery disease [15,16].

From a surgical standpoint, Wilson et al. reported that 8.3% of patients who required non-cardiac surgery within 60 days of coronary stenting underwent gastrointestinal or abdominal surgery [17]. Tokushige et al. reported that patients undergoing PCI frequently underwent abdominal surgery or gastrointestinal endoscopic procedures within a few years [18].

Thus, this suggests that there is a significant association between gastrointestinal malignancies and coronary artery disease.

### 4.2. Non-Cardiac Surgery and Coronary Revascularization

The risk of cardiovascular events in the perioperative period of non-cardiac surgery increases in the presence of IHD [19,20]. However, prophylactic revascularization before major vascular surgery for all patients with stable coronary disease does not improve the perioperative period or the long-term clinical course [21,22]. In addition, preoperative revascularization in patients with stable IHD before noncardiac surgery is not beneficial, as it does not reduce perioperative mortality or the incidence of acute myocardial infarction [23]. Regardless of whether IHD or malignancy is detected earlier, it is reasonable to assume that if invasive treatment for malignancy is required, it should be prioritized before revascularization for stable IHD.

Although ACS is preceded by revascularization, the risk of cardiovascular events in the perioperative period of non-cardiac surgery is particularly high within the first month after onset and decreases over time [24,25,26,27,28]. Surgical delays are desirable when malignancy is detected shortly after myocardial infarction; however, appropriate surgery may still be feasible with adequate discussion between the cardiologist and oncologist. Although cardiac death accounts for the majority of fatalities in the first month after the onset of ACS, non-cardiac disease accounts for two-thirds of deaths, with malignancy accounting for a particularly large proportion [29]. The detection of malignancy is also important in patients with ACS since long-term prognosis is often determined by non-cardiac disease.

### 4.3. Antiplatelet Therapy and Bleeding Complications

Since the efficacy of DAPT in preventing stent thrombosis has been established, it is now the standard of care after PCI [30]. However, antiplatelet therapy carries a risk of increased bleeding complications, and DAPT is associated with a 7.4-fold increase in the risk of upper gastrointestinal bleeding [31].

Active malignancies are one of the high-risk factors for bleeding [32,33,34]. Gastric and colorectal cancers are the major causes of cancer-related death by organ [35], and these gastrointestinal malignancies are often clinically detected following gastrointestinal bleeding during antiplatelet therapy [9]. Although no major bleeding complications were observed in this study, the lower hemoglobin levels in patients with malignancies may have reflected minor bleeding. Cancer complications increase the risk of such bleeding complications and cardiac death, and the dose and duration of DAPT could be considered [10]. On the other hand, DAPT might need to be discontinued during endoscopic or surgical treatment of gastrointestinal malignancies, which might increase the risk of stent thrombosis. In this context, it is reasonable to assess for malignancy before PCI in CCS. Even if DAPT has already been introduced after ACS, endoscopy without biopsy does not necessitate DAPT withdrawal [36]. Considering the aforementioned risk of bleeding complications, performing endoscopy to determine the treatment strategy is advisable, even in patients with ACS.

### 4.4. FIT and Malignancy

FIT is a well-established strategy for colorectal cancer screening in the general population [11,12]. The detection rate of colorectal cancer by FIT is comparable to that of endoscopy [12], and should be favored as an initial test due to its simplicity and non-invasive nature. On the other hand, FIT may yield positive results for upper gastrointestinal hemorrhage only in cases of massive hemorrhage. However, the positive rate of FIT is low because hemoglobin is often degraded by proteases in gastric and pancreatic juices or denatured by other digestive juices, reducing its antigenic properties. In this regard, the usefulness of FIT for detecting upper gastrointestinal bleeding remains controversial. On the other hand, endoscopy is highly beneficial, because biopsy allows histological diagnosis and thus treatment decisions, and it is widely used as a screening for gastrointestinal diseases. The diagnostic significance of performing upper and lower gastrointestinal endoscopy in the perioperative period of PCI with DAPT is evident, considering that cancers at risk for bleeding can be detected even in FIT-negative cases in this study. Although this study did not examine ancillary screening methods such as tumor markers or imaging modalities, combining these with FIT or endoscopy may improve detection of gastrointestinal malignancies.

### 4.5. Potential Confounding Factors Associated with Survival of PCI Patients with Malignancy

In this study, the survival rate in PCI patients with gastrointestinal malignancy was low, although the limited number of patients prevented a statistical analysis for confounding factors. As noted above, the acute phase of cardiac disease is likely to affect prognosis, but later, the malignancy itself and treatment-related complications (e.g., infection, immunodeficiency, bleeding or anemia, and thromboembolism) might play a role in survival in cancer patients [29]. Alternatively, classical risk factors common to cardiovascular disease, such as oxidative stress and chronic inflammation, might be strongly expressed and affect systemic status [13]. Collaboration between cardiologists and oncologists about patient status, treatment strategies, and possible adverse events could lead to early response to critical complications.

### 4.6. Clinical Significance of This Study

This study is the first to investigate the prevalence and factors of malignancy through upper and lower gastrointestinal endoscopy in PCI’s perioperative period.

Although the detection rate of malignancy by endoscopic screening in the general population varies, a review by Yashima et al. showed that the detection rate of gastric cancer by screening endoscopy in Japan or Asia ranged from 0.30 to 0.87% [37]. Also, Toyoshima et al. reported a colorectal cancer detection rate of 0.9% in patients indicated for endoscopy in Japan, including screening and surveillance as well as FIT-positive cases [38]. Compared to these reports, more gastrointestinal malignancies were detected in PCI patients of this study.

In patients with ACS, endoscopy was performed promptly after acute revascularization once hemodynamic stability was achieved. Early malignancy detection allows timely discussions between cardiologists and oncologists for treatment planning. Patients with CCS, treated on a standby basis, have the advantage of undergoing endoscopy before coronary intervention, facilitating early management of malignancy or hemorrhagic lesions. No complications were associated with endoscopy.

Although FIT provides an opportunity to detect malignancies, it does not identify all malignancies or gastrointestinal bleeding. Therefore, gastrointestinal endoscopy should be performed whenever possible, including in ACS cases. Notably, despite detection without bleeding complications in the perioperative period of PCI, malignancy prognosis remains poor, irrespective of treatment.

### 4.7. Limitations

First, this was a retrospective, observational study conducted at a single institution in Japan with a relatively small sample size. Therefore, the number of patients with malignancy was limited, which could limit statistical power and make it difficult to generalize results to other races with different genetic and dietary risk factors for gastrointestinal malignancies. Also, it was difficult to examine factors affecting outcomes in patients with gastrointestinal malignancy. Further, the lack of a true control group without PCI limits the ability to determine if the prevalence of malignancies is higher than in the general population. Second, a total of 108 patients were excluded from the study, including patients who did not consent to upper or lower endoscopy or whose general condition precluded the examination, which may introduce selection bias. The prevalence of malignancy in PCI patients may be lower if the number of patients with malignancy among the excluded patients is extremely small. Validations in more rigorous multicenter prospective studies are desirable. Third, the extent of coronary artery disease (number of vessels involved, degree of stenosis, etc.), as well as the number of stents implanted, SYNTAX score, and TIMI risk score as surrogate markers were not considered, making it difficult to assess the severity of IHD. This may affect the duration of antiplatelet therapy and the risk posed by malignancy. Also, the timing of DAPT and gastrointestinal evaluation was left to the discretion of the attending physician in line with clinical practice, and the association between antiplatelet drug administration and malignancy detection was not clear. In addition, FIT was not performed in many cases, particularly in CCS cases. Fourth, although gastric cancer or gastric/duodenal ulcers were clearly associated with *Helicobacter pylori* carriage, we were unable to assess the presence of *Helicobacter pylori* carriage in all patients in our study. Furthermore, tumor marker measurements that suggested the presence of gastrointestinal malignancies were not performed. Finally, it would have been more meaningful to compare survival in the group that was not endoscoped and excluded from the study (n = 108), the group that was endoscoped and cancer-free (n = 363), and the group that had cancer (n = 30); unfortunately, this study could not analyze the survival of the first two groups.

## 5. Conclusions

A high proportion of Japanese patients undergoing PCI had gastrointestinal malignancies, regardless of whether they had ACS or CCS, and their prognosis was poor. Gastrointestinal endoscopic evaluation in the perioperative period of PCI could detect malignancy without complications and might lead to appropriate cancer treatment.

## Figures and Tables

**Table 1:** Patient characteristics.

Variables	All (n = 393)	Malignancy (n = 30)	No Malignancy (n = 363)	*p*
Age, year	75 (66–82)	74 (71–83)	75 (65–82)	0.2266
Male, n (%)	277 (70)	23 (77)	254 (70)	0.4398
Body surface area, m^2^	1.64 ± 0.19	1.61 ± 0.18	1.64 ± 0.19	0.3254
Acute coronary syndrome, n (%)	142 (36)	10 (33)	132 (36)	0.7398
Heart failure, n (%)	89 (23)	9 (30)	80 (22)	0.3167
Hypertension, n (%)	299 (76)	24 (80)	275 (76)	0.6006
Diabetes mellitus, n (%)	159 (40)	12 (40)	147 (40)	0.9576
Dyslipidemia, n (%)	227 (58)	14 (47)	213 (59)	0.2005
Atrial fibrillation/flutter, n (%)	68 (17)	7 (23)	61 (17)	0.3636
Smoking, n (%)	219 (56)	15 (50)	204 (56)	0.5113
Hemoglobin, g/dL	12.7 ± 1.9	12.1 ± 2.2	12.7 ± 1.9	0.0649
Albumin, g/dL	3.7 (3.4–4.0)	3.5 (3.3–3.7)	3.7 (3.4–4.0)	0.0373
Sodium, mEq/L	141 (139–143)	141 (139–142)	141 (139–143)	0.0951
eGFR, mL/min/1.73 m^2^	58.9 (45.7–72.8)	53.1 (41.6–70.7)	59.1 (46.0–73.1)	0.4676
BNP, pg/mL	54.6 (21.7–154.3)	79.6 (39.9–190.0)	53.5 (21.5–154.0)	0.5312

Data are expressed as mean ± standard deviation, median (IQR), or n (%). eGFR, estimated glomerular filtration rate; BNP, brain natriuretic peptide.

**Table 2:** Progression of malignancies and treatments performed.

Malignancy by Organ	Stage	Treatment
Colorectal cancer, n = 18	Early, n = 11	Endoscopy, n = 7 Surgery, n = 3 Supportive, n = 1
	Advanced, n = 6	Surgery, n = 6
	Unknown, n = 1	-
Gastric cancer, n = 8	Early, n = 6	Endoscopy, n = 3 Surgery, n = 1 Supportive, n = 2
	Advanced, n = 2	Surgery, n = 1 Chemotherapy, n = 1
Esophageal cancer, n = 2	Advanced, n = 2	Endoscopy, n = 1 Chemoradiation, n = 1
Laryngeal cancer, n = 1	Unknown, n = 1	-
Overlap of gastric and colorectal cancer, n = 1	Early, n = 1	Endoscopy for both, n = 1

**Table 3:** Multivariate analysis for malignancy detection.

Variables	OR (95% CI)	*p*
Age	1.00 (0.94–1.06)	0.9338
Albumin	1.19 (0.32–4.34)	0.7960
FIT	12.36 (3.51–43.5)	<0.0001

OR, odds ratio; CI, confidence interval; FIT, fecal immunochemical testing.

## Data Availability

The deidentified participant data will not be shared.

## Abbreviations

The following abbreviations are used in this manuscript:

ACS	acute coronary syndrome
CCS	chronic coronary syndrome
DAPT	dual antiplatelet therapy
FIT	fecal immunochemical testing
IHD	ischemic heart disease
PCI	percutaneous coronary intervention
